# Renal function is a determinant of subjective well-being in active seniors but not in patients with subjective memory complaints

**DOI:** 10.1186/1756-0500-7-647

**Published:** 2014-09-15

**Authors:** Lovisa A Olsson, Nils-Olof Hagnelius, Torbjörn K Nilsson

**Affiliations:** Department of Laboratory Medicine/Clinical Chemistry, Örebro University Hospital, Södra Grev Rosengatan, 701 85 Örebro, Sweden; School of Health and Medical Science, Örebro University, Fakultetsgatan 1, 70281 Örebro, Sweden; Department of Geriatrics, Örebro University Hospital, Södra Grev Rosengatan, 701 85 Örebro, Sweden; Department of Medical Biosciences, Clinical Chemistry, Umeå University, Byggnad 6M, 90185 Umeå, Sweden

**Keywords:** Creatinine, Cystatin C, Subjective well-being, Biomarkers, Elderly, Renal function

## Abstract

**Background:**

During our whole life span, factors influencing health and functioning are accumulated. In chronic kidney disease, quality of life is adversely affected. We hypothesized that biomarkers of renal function could also be determinants of subjective well-being (SWB) in Swedish elderly subjects. SWB was assessed by the Psychological General Well-Being index (PGWB index) in two study groups: Active seniors (AS) consisted of community-dwelling elderly Swedes leading an active life (*n* = 389), and the DGM cohort (*n* = 300) consisted of subjects referred to the Memory Unit at the Department of Geriatrics for memory problems, Serum creatinine, cystatin C, and eGFR (CKD-EPI) were used as biomarkers of renal function.

**Results:**

There were no significant differences in cystatin C and eGFR values between the two cohorts: cystatin C medians 0.88 vs 0.86 mg/L and eGFR 73 vs 80 mL/min/1.73 m^2^ (AS vs DGM). In the AS cohort cystatin C was negatively related to PGWB index in women (*P <* 0.001*, R*^*2*^ *≈* 5%), and the covariates age and BMI did not improve the models. The renal biomarkers were unrelated to the PGWB index in the DGM cohort. Cystatin C in the AS cohort was adversely related to the PGWB subdimensions anxiety, depressed mood, positive well-being, and vitality in women, but in men only to depressed mood (*P* < 0.006; *R*^2^ *≈* 6%). In the DGM cohort, depressed mood in men was also significantly related to cystatin C (*P* = 0.050), but not in women.

**Conclusions:**

Renal function even within the normal range, measured by serum cystatin C concentration, has significant and sex specific associations with subjective well-being and its subdimensions in healthy elderly subjects. Maintenance of good renal function in aging may be of importance in maintaining a high subjective well-being.

## Background

During our whole life span, factors that influence health and functioning are accumulated; these factors include genetics, environment, and individual life experience and exposures [1]. Moderate renal dysfunction is increasing worldwide [2] and has been associated with an increased risk of death from cardiovascular events and kidney failure [3–5] and a number of other clinical conditions [6, 7] *.* While it is well known that in more pronounced renal dysfunction (chronic kidney disease, CKD), quality of life (QoL) is adversely affected [8–10] there is a relative dearth of studies addressing the possibility that CKD could also be related to Subjective Well-Being (SWB), a person’s own evaluation of his or her life. Such evaluations may be judgments about the person’s life as a whole or evaluations of specific dimensions of life, both positive and negative [1, 11]. In particular, next to nothing is known about the possibility that SWB (or QoL) might be affected even by moderate renal dysfunction (MRD), perhaps even while biomarkers of renal function, used to estimate glomerular filtration (eGFR), still remain within the reference interval.

S-creatinine and S-cystatin C are commonly used as biomarkers to calculate eGFR. Among the disadvantages of C-creatinine as a GRF marker are its dependence on muscle mass, age, race and sex of the subjects [12], tubular secretion, drugs and dietary intake [13, 14]. Cystatin C is a protease inhibitor produced in nearly all human cells. Its serum concentration is independent of muscle mass and sex [12], and it has been proposed as a better biomarker of GFR than creatinine [15].

We hypothesized that a possible negative effect of kidney function on SWB could contribute substantially to the total variance in SWB in an elderly population even in the absence of CKD. The aim of this study was thus to look for possible associations between established biomarkers of renal function (S-creatinine, S-cystatin C, and eGFR) and subjective well-being in a sample of 689 Swedish elderly subjects.

## Methods

### Subjects

**Figure 1:**
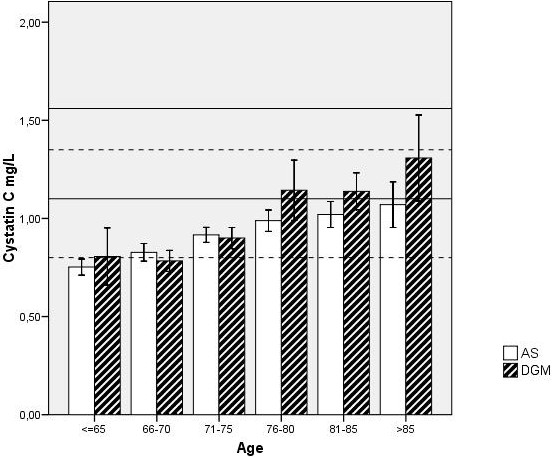
**Cystatin C values in in the two study groups stratified by age in 5-year intervals.** Mean values and 95% CIs of the means are shown. The dotted lines show our in-house reference values for the 51-65 year-olds (95% CI), and the continuous lines those of the 65-80 year-olds.

*Active Seniors (AS)* cohort was recruited during 2003 and 2004 by a multi-phase sampling procedure aimed at an elderly retired population living in various communities in Central Sweden. The locations for the recruitment were selected to represent a broad range of socioeconomic levels and included rural as well as urban and suburban areas. The sample consisted of 389 senior citizens and was recruited from several retired persons’ organisations, which implicates that they are independent and socially active. Being retired, living independently in their own homes in addition to participation in such organisations were the sole inclusion criteria, not pre-set health criteria. We have designated them as ‘Active Seniors’ in contrast to elderly persons that do not engage themselves in such social activity*.* All were Caucasians, most of them born in the 1920’s and 1930’s, mean age at sampling was 74 ± 5 years for both sexes and the sex ratio M/F was 127/262. Based upon the cystatin C values in different age groups (Figure 1), the number of AS subjects with cystatin C above the upper reference limit (1.55 mg/L) was 7 women and 3 men (total prevalence 2.6%).

A random subset of the AS cohort was assessed by the Mini Mental State Examination (MMSE) [16] and the clock drawing test (CDT) judged according to Shulman [17]. These subjects (n = 154) were found to be cognitively intact, defined as MMSE ≥ 28 and CDT ≥ 4 Neither the PGWB score nor any of the studied biomarkers differed significantly between this subgroup and the rest of the AS study group. We therefore regard the AS cohort as a cognitively intact group of elderly subjects.

*S*enior subjects with *subjective or objective cognitive complaints* (DGM cohort) were recruited from an incident case study at the University Hospital, Örebro Sweden [18]. Briefly this study population consisted of 300 consecutive patients (143 men and 157 women) who were referred to the Memory Unit at the Department of Geriatrics for diagnostic assessment and treatment of suspected cognitive problems. The inclusion period extended from May 2003 to August 2007. The cognitive problems had to be mild or moderate, defined as MMSE score ≥10; according to current Swedish research ethics legislation it is unethical and thus forbidden to include subjects with more severe dementia due to their reduced autonomy. Dementia diagnoses were based on *DSM-IV* criteria ICD-10 criteria was used to divide dementia patients into different diagnostic categories. Mixed dementia (F 00.2 according to ICD-10) was diagnosed in cases of coexistence of AD and a history of vascular risk factors e.g. transitory ischemic attack(s). atrial fibrillation, hypertension, diabetes mellitus, Hachinski ischemic score ≥ 6 and CT scan showing ischemic changes. Probable AD was diagnosed in accordance with the NINCDS-ADRDA criteria. Of the 73 subjects classified as ND, 39 were MCI. Everyone in the study group completed the PGWB questionnaire without assistance during their visit at the Memory unit. Based upon the cystatin C values in different age groups (Figure 1), the number of DGM subjects with cystatin C above the upper reference limit (1.55 mg/L) was 12 women and 9 men (total prevalence 7%).

All subjects gave a specific and written informed consent to the present study including genotyping and biobanking of the donated samples. The Research Ethics Committee of Örebro County Council and the Regional Ethics Review Board (Uppsala) approved the study. Height and weight (SECA digital scale, 99% of the subjects) were measured and BMI (kg/m2) was calculated. The resting systolic and diastolic blood pressure was measured using an automatic oscillometric method (Dinamap model XL Critikron, Inc., Tampa, Florida.). The equipment has been validated.

### Assessment of subjective well-being

The Psychological General Well-Being (PGWB) index, accessible from MAPI Research institute, http://www.mapi-trust.fr, was used to measure subjective well-being or distress [19]. It consists of 22 items that reflect a sense of subjective well-being and distress during the past week. The items are divided into six dimensions, anxiety, depressed mood, positive well-being, self-control, general health, and vitality; these are also combined to a global overall score of SWB. Response categories for all items were simplified to a 6-point Likert scale. A high value indicates a high level of well-being. In order to achieve this interpretation of the score-points even for the dimensions which reflect distress (depressed mood and anxiety). score-points for these items were assigned in the direction of “6” to “1” in contrast to the usual direction of “1” to “6”. The overall sum of scores gives a maximum value of 132 (best SWB) and a minimum of 22 (poorest SWB).

### Blood sampling and biochemical measurements

Blood samples were taken with the subjects in the supine position, by venipuncture using vacuum tubes with gel, Becton Dickison. Serum was obtained after clotting for 30 to 60 minutes at room temperature and centrifuging at room temperature for 10 minutes at 2000 g. All samples were stored at -80°C. The serum samples were analysed on a Hitachi 911 multianalyser, Roche, Mannheim, FRG. Creatinine was analysed using a enzymatic method, Crea plus, from Roche/Boehringer Mannheim FRG, and the calibrator was IDMS standardised. Albumin, IgG, IgA and IgM were analysed by immunoturbidimetry using reagents from DAKO, Copenhagen, Denmark and cystatin C by immunoparticle turbidimetry from Gentian AS, Moss, Norway. LDL and HDL cholesterol were measured by direct, homogeneous assays based on detergent treatment of the serum. N-geneous™ HDL-c and N-geneous™ LDL reagents, respectively, from Genzyme Corporation, Cambridge, MA, USA.

Estimated glomerular filtration rate (eGFR) was calculated using the equation which include both creatinine and cystatin C from the Chronic Kidney Disease Epidemiology collaboration. This equation includes both creatinine and cystatin C as well as age, sex and race [20]. In the present study all participants were of European descent.

### Statistics

All variables were checked for normality of distribution and collinearity before analysis. Continuous variables with a skewed distribution were transformed using natural logarithms before regression analyses were made. The Mann-Whitney rank sum test was used to compare the PGWB sum and PGWB sub-dimension between gender in the two cohorts, median and interquartile range for descriptive statistics of these variables.

A forward multiple regression was used to determine the degree to which variance in PGWB index was explained by age, BMI, gender and, cystatin C or creatinin. To examine which of the subdimensions contributed to the variance in PGWB index ANCOVA analyses were done with age, BMI, and biomarkers of kidney function as covariates. All multivariate regression models were performed after stratification, since we have found profound differences between SWB in the AS and DGM cohorts [21].

Statistical analyses were performed using SPSS version 15.0 (Chicago, Illinois, USA), and PGWB Statistical significance were considered with a probability value <0.05.

In the AS study group, 4 subjects failed to complete the PGWB questionnaire and responses from 12 subjects failed to answer one or two questions randomly distributed among the 22 questions. The missing answers were imputed using the manual from MAPI research institute.

## Results

### Baseline characteristics

Baseline characteristics of the two study groups are shown in Table 1. Men in the DGM cohort were significantly younger than in the AS cohort. Within the AS cohort men had significantly higher diastolic blood pressure and serum creatinine concentrations than women. Within the DGM cohort creatinine and plasma IgA differed significantly between sexes. Serum creatinine mean concentrations also differed between women in the AS and DGM cohorts, while there was no difference between any sub-group comparison concerning cystatin C mean concentrations. There was a difference in eGFR between the two study groups, and also between the sexes in the AS cohort, but the prevalence of subjects with an eGFR (KDGIO) < 60 mL/min/1.73 m^2^ was similar, 22%, in both the AS and DGM cohorts. A cystatin C concentration >1.1 mg/mL was found in 16% of subjects in the AS cohort and 22% in the DGM cohort.

**Table 1:** **Baseline and laboratory characteristics of the studied 689 seniors**

Mean (SD) and p-values for the sex differences are shown	Active seniors cohort			DGM cohort				
	Women n = 262	Men n = 127	***P*** ^a^	Women n = 157	Men n = 143	***P*** ^a^	***P*** ^b^	***P*** ^c^
Age. yr	74.4 (7.2)	75.0 (6.4)	NS	73.3 (10.6)	72.5 (9.5)	NS	NS	0.028
Height, cm	163 (5.8)	177 (6.7)	<0.001	163 (5.9)	176 (1.2)	<0.001	<0.001	<0.001
BMI, kg/cm2	26.0 (4.2)	26.1 (3.5)	NS	25.8 (3.7)	26.2 (3.7)	NS	NS	NS
systBPsitting, mm Hg	148.9 (26.1)	146 (22.5)	NS	150 (23.7)	149 (23.6)	NS	NS	NS
diastBPsitting, mm Hg	76.3 (11.3)	78.0 (10.4)	0.013	82.3 (12.5)	84.1 (12.1)	NS	<0.001	<0.001
S-Creatinine, umol/L	87.4 (21.5)	101 (16.8)	<0.001	78.9 (36.6)	97.9 (58.9)	<.001	<0.001	NS
S-Cystatin C, mg/L	1.02 (0.25)	1.04 (0.22)	NS	0.98 (0.54)	1.01 (0.45)	NS	NS	NS
eGFR mL/min/1,73 m^2^	70.1 (15.95)	74.0 (14.48)	0.022	76.1 (22.8)	78.6 (23.24)	NS	0.002	0.049
hS-CRP, mg/L	2.06 (2.13)	2.90 (3.74)	NS	3.34 (6.38)	5.51 (16.01)	NS	NS	NS
P-Albumin, g/L	40.9 (3.41)	40.7 (3.46)	NS	36.5 (3.30)	36.9 (3.22)	NS	<0.001	<0.001
P-IgG, g/L	11.9 (2.7)	12.1 (2.9)	NS	9.83 (2.38)	10.24 (2.69)	NS	<0.001	<0.001
P-IgM, g/L	1.23 (0.79)	1.09 (1.50)	NS	1.01 (0.99)	0.86 (0.62)	NS	0.035	NS
P-IgA, g/L	2.37 (.08)	1.40 (0.36)	0.004	2.16 (1.21)	2.62 (1.07)	0.002	NS	NS

eGFR = CKD-EPI creatinin-cystatin C equation [20].

*P* ^a^ Sex difference within cohort.

*P* ^b^ Difference between women in the AS and DGM cohorts.

*P* ^c^ Difference between men in the AS and DGM cohorts.

There were group differences in SWB, assessed by the PGWB instrument, between the two cohorts, see Table 2. In the DGM cohort men had significantly higher well-being than women in all sub-dimensions but positive well-being. In the AS cohort the only sex difference was that women had a significantly lower degree of self-control. When comparing women in the AS and the DGM cohorts, DGM women had significantly lower SWB than AS women. That was also the finding comparing men in both cohorts, except for general health.

**Table 2:** **SWB as PGWBsum and its subdimensions in the study groups active seniors and DGM**

	Active seniors cohort			DGM cohort				
	Women	Men	***P*** ^a^	Women	Men	***P*** ^a^	***P*** ^b^	***P*** ^c^
Anxiety	27 (24-29)	27 (25-29)	Ns	22 (20-27)	25 (22-28)	0.005	<0.001	<0.001
Depressed mood	17 (15-18)	17 (15-18)	NS	15 (13-17)	16 (14-17)	0.019	<0.001	<0.001
Pos.well-being	18 (15-19)	18 (16-19)	NS	15 (12-18)	16 (13-18)	Ns	<0.001	<0.001
Self control	15 (16-17)	17 (16-17)	0.018	14 (12-16)	15 (13-16)	0.006	<0.001	<0.001
General. health	16 (14-17)	16 (13-17)	NS	14 (12-13)	15 (13-17)	0.006	<0.001	NS
Vitality	19 (17-21)	19 (17-21)	NS	17 (13-20	18 (16-20)	0.016	<0.001	0.003
PGWB sum	112 (101-118)	113 (105-118)	NS	97.5 (85-109)	104 (90-115)	0.001	<0.001	<0.001

*P* ^a^ Sex difference within cohort.

*P* ^b^ Difference between women in the AS and DGM cohorts.

*P* ^c^ Difference between men in the AS and DGM cohorts.

Median and the interquartile range 25% -75% are shown.

### Biomarkers of renal function and PGWB index

In the AS cohort, cystatin C was significantly and negatively related to PGWB index in women, explaining ≈ 5% of the variance in SWB, as seen in Table 3. Addition of the covariates age and BMI did not substantially improve this model. In men too there was a negative correlation of cystatin C with PGWB index, the β coefficient was numerically similar to the one in women (-10.1 vs. -12.2), and reached borderline statistical significance (*P* = 0.06). Just as in women, addition of age and BMI as covariates did not change this pattern; cystatin C retained its β value around the same numerical level (-10.7), again showing borderline significance (*P* = 0.07).

**Table 3:** **ANCOVA models examining the association of the PGWB index with the covariates cystatin C, creatinine, eGFR, BMI, and age**

AS cohort	Predictor variables	β	Women		β	Men	
Model			***P***	***R*** ^2^model		***P***	***R*** ^2^model
1	cystatin C	-12.2	**<0.001**	0.048	-10.1	0.061	0.019
2	Age	-0.341	**0.006**	0.030	-0.013	0.942	-0.008
	BMI	0.262	0.206		-0.315	0.345	
3	Age	-0.201	0.125	0.053	0.119	0.540	0.009
	BMI	-0.167	0.418		-0.150	0.662	
	cystatin C	-9.721	**0.008**		-10.69	0.071	
4	creatinine	-10.359	**0.017**	0.019	-7.592	0.318	0.00
5	Age	-0.283	**0.029**	0.058	0.020	0.915	-0.009
	BMI	-0.287	0.166		-0.281	0.406	
	creatinine	-8.31	0.058		-8.255	0.288	
4	eGFR	0.164	**0.002**	0.019	0.128	0.116	0.009
5	Age	-0.203	0.157	0.038	0.160	0.455	0.003
	BMI	-0.198	0.341		-0.165	0.633	
	eGFR	0.113	0.070		0.156	0.104	
**DGM cohort**	**Predictor variables**	**β**	**Women**		**β**	**Men**	
**Model**			***P***	***R*** ^**2**^ **model**		***P***	***R*** ^**2**^ **model**
1	Cystatin C	0.42	0.856	-0.006	-1.4	0.651	-0.006
2	Age	0.045	0.709	-0.009	0.136	0.366	-0.007
	BMI	0.274	0.429		0.258	0.501	
3	Age	0.045	0.728	-0.016	0.266	0.116	0.00
	BMI	0.250	0.475		0.332	0.394	
	cystatin C	0.187	0.938		-3.699	0.289	
4	creatinine	2.162	0.590	-0.005	2.943	0.526	-0.004
5	Age	0.035	0.774	-0.014	0.115	0.473	-0.013
	BMI	0.271	0.434		0.261	0.498	
	creatinine	2.016	0.622		1.969	0.691	
6	eGFR	0.021	0.698	-0.016	-0.016	0.787	0.000
7	Age	0.113	0.457	-0.012	0.279	0.173	-0.005
	BMI	0.274	0.433		0,312	0.423	
	eGFR	0.050	0.462		0.053	0.514	

Numbers in boldface indicate statistically significant associations.

In the AS cohort, serum creatinine too was, in women, significantly and negatively related to PGWB index (*P* = 0.02) but explained only ≈ 2% of its variance. Adding the covariates age and BMI increased the explanatory power of the model to ≈ 6%. In men, creatinine was unrelated to PGWB index, and adding age and BMI as covariates did not improve the insignificant *R*^2^ (Table 3, models 4 and 5).

In contrast, in the DGM cohort neither cystatin C nor creatinine were significantly related to PGWB index, and none of the extended models had any explanatory power (Table 3, lower panel).

### Cystatin C and PGWB subdimensions

In Table 4, the relations of PGWB subdimensions with cystatin C are shown. In women in the AS cohort, cystatin C was significantly and adversely related to the dimensions anxiety, depressed mood, positive well-being, and vitality. In contrast, in women in the DGM cohort cystatin C was unrelated to any of the PGWB subdimension.

**Table 4:** **ANCOVA of the PGWB subdimensions**

Women	Predictor variables	Active seniors cohort			DGM cohort		
PGWB subdimension		β	***P***	***R*** ^2^model	β	***P***	***R*** ^2^model
Anxiety	Age	-0.012	0.728	0.010	0.046	0.196	-0.003
	BMI	0.019	0.730		0.071	0.462	
	cystatin C	-1.955	**0.046**		0.265	0.692	
Depressed mood	Age	-0.024	0.260	0.026	-0.029	0.183	0.001
	BMI	0.002	0.948		0.055	0.353	
	Cystatin C	-1.269	**0.029**		0.085	0.865	
Positive well-being	Age	-0.039	0.191	0.031	-0.15	0.566	-0.003
	BMI	-0.001	0.975		0.089	0.205	
	Cystatin C	-1.886	**0.023**		-0.162	0.738	
Self control	Age	-0.024	0.259	0.018	0.012	0.604	-0.010
	BMI	-0.014	0.680		0.046	0.461	
	Cystatin C	-1.019	0.078		0.303	0.83	
General health	Age	-0.070	**0.005**	0.073	0.005	0.854	-0.015
	BMI	-0.078	**0.043**		-0.054	0.424	
	Cystatin C	-1.021	0.133		0.037	0.938	
Vitality	Age	-0.031	0.331	0.064	0.026	0.462	-0.015
	BMI	-0.093	0.063		0.043	0.652	
	Cystatin C	-2.609	**0.003**		-0.339	0.606	
**Men**	**Predictor variables**	**Active seniors cohort**			**DGM cohort**		
**PGWB subdimension**		**β**	***P***	***R*** ^**2**^	**β**	***P***	***R*** ^**2**^
Anxiety	Age	-0.033	0.496	-0.015	0.069	0.122	-0.002
	BMI	0.037	0.670		0.093	0.365	
	cystatin C	-1.420	0.335		-0.567	0.537	
Depressed mood	Age	-0.22	0.462	0.063	0.041	0.134	0.011
	BMI	-0.004	0.932		0.038	0.552	
	Cystatin C	-2.472	**0.006**		-1.122	**0.050**	
Positive well-being	Age	-0.014	0.728	0.025	0.025	0.478	-0.12
	BMI	0.015	0.828		0.070	0.392	
	Cystatin C	-2.649	**0.031**		0.154	0.833	
Self control	Age	0.072	0.009	0.032	0.060	**0.032**	0.032
	BMI	0.027	0.572		0.073	0.256	
	Cystatin C	-1.237	0.135		-0.944	0.103	
General health	Age	0.009	0.844	0.029	0.028	0.357	-0.006
	BMI	-0.145	0.059		-0.012	0.869	
	Cystatin C	-1.654	0.209		-0.871	0.171	
Vitality	Age	0.034	0.480	-0.007	0.042	0.240	-0.009
	BMI	-0.050	0.554		0.069	0.398	
	Cystatin C	-1.560	0.283		-0.349	0.635	

Numbers in boldface indicate statistically significant associations.

In men, in the AS cohort there was a significantly adverse relation between cystatin C and depressed mood, which explained ≈ 6% of its variance (*P* = 0.006). In the DGM cohort too, cystatin C in men was significantly and adversely correlated with depressed mood (*P* = 0.050), although the variance explained was minor (≈ 1%). In the AS cohort, there was also an adverse relation between positive well-being and cystatin C (*P* = 0.03), explaining ≈ 2.5% of that subdimension.

## Discussion

The main finding was a negative correlation between SWB, assessed by the PGWB index and serum cystatin C as a biomarker of renal function, even after adjusting for age and BMI, in Active Seniors but not in subjects with subjective memory complaints (DGM cohort), two cohorts of Swedish elderly subjects with a low prevalence of chronic kidney disease (CKD) as judged from their serum cystatin C concentrations.

There were marked gender differences in the adverse relation between cystatin C and the subdimensions of SWB. In women in the AS cohort all subdimensions except self control were adversely related to cystatin C, whereas in the DGM cohort cystatin C was unrelated to any SWB subdimension. (see Table 4). In contrast, in men, there was a significant adverse relation of cystatin C only with depressed mood, and this relation was seen both in the AS and in the DGM cohorts. Interestingly, in the AS cohort the beta factor for men was twice that of women (-2.5 vs. -1.3) somehow reflecting a higher sensitivity to depressed mood in men, further supported by the fact that this adverse relation was significant even in males in the DGM cohort (beta value -1.1).

To the best of our knowledge, the above findings regarding the adverse relations between a biomarker of renal function and important aspects of subjective well-being in subjects with renal function within the normal range have not been reported before. Previous studies about QoL have mostly been conducted in patients with CKD, end state renal disease (ESRD), or on hemodialysis, with QoL as outcome variable, not SWB. Health Related Quality of Life (HRQoL) has been found to be reduced in proportion to the severity of CKD [22, 23]. Our findings thus extend the scope of renal function twofold, both to another dimension besides QoL, namely SWB, and to the importance of minor reduction in renal function as a determinant of SWB.

In the Health, Aging and Composition study, cystatin C was not associated with depression at baseline but participants having cystatin C higher than 1.25 mg/L had an increased risk to develop diagnosed depression [24]. In the prospective Cardiovascular Health Study, cystatin C was associated with depression among participants without CKD at baseline but this was a borderline association after adjusting for both demographic and clinical measures [25]. We regard these studies as consistent with our finding of an adverse relation between cystatin C and depressed mood in both sexes in the AS cohort, and among men even in the DGM cohort.

SWB includes a person’s evaluation of his or hers life, both positive and negative feelings as well as perceptions of one’s situation. In our model cystatin C explained 6% of the SWB variance which is regarded as clinically relevant. SWB has been associated with longevity [26–28]. Chida and Steptoe showed in their meta-analysis that high SWB was associated with reduced mortality in healthy populations [29]. They also concluded that a high SWB appeared to be protective against increased mortality, even in CKD patients. We speculate that the association between SWB and renal function could provide a missing link between SWB, longevity, and mortality [3, 4, 30] even in subjects without CKD.

SWB differs between the sexes [31, 32] as also shown in our cohorts. Therefore it is of special relevance that a number of renal functions also differ between the sexes, further supporting a link between renal function and SWB. [33, 34]. We contend that, based on the documented links between renal function, SWB, and mortality, monitoring of cystatin C in the elderly may help identifying subjects in need of more proactive treatment to prevent further deterioration of renal function. Successful intervention might impact both SWB and mortality positively in the elderly. Prospective studies will be necessary to address this possibility.

There are some limitations in our study. SWB is a function of many variables such as socioeconomic status, marital status, physical health, life expectations [35–37], and in this study we were not able to control for all of these factors. However, reverse causation (low PGWB scores due to poor SES and/or marital status causing a reduced GFR) seems unlikely as confounder, since establishment of marital status and SES regularly take place early in life whereas reduced GFR develops gradually in the aging subject (Figure 1), and it thus appears unlikely that a reduced GFR would significantly affect marital status and SES. Personality and genetic variation may also contribute substantially [38]. These factors should be included in future studies.

## Conclusion

Renal function even within the normal range, assessed by serum cystatin C concentration, has significant and sex specific associations with SWB and its subdimensions. Maintenance of good renal function in aging may therefore be of importance not only in preventing somatic deterioration in old age but also in maintaining a good subjective well-being.
